# Hyperglycemia Induced by Glucokinase Deficiency Accelerates Atherosclerosis Development and Impairs Lesion Regression in Combined Heterozygous Glucokinase and the Apolipoprotein E-Knockout Mice

**DOI:** 10.1155/2016/8630961

**Published:** 2016-09-28

**Authors:** Damilola D. Adingupu, Suvi E. Heinonen, Anne-Christine Andréasson, Mikael Brusberg, Andrea Ahnmark, Margareta Behrendt, Brendan Leighton, Ann-Cathrine Jönsson-Rylander

**Affiliations:** ^1^CVMD iMED, AstraZeneca R&D, Gothenburg, Sweden; ^2^The Research Network, Sandwich, UK

## Abstract

*Aim*. Models combining diabetes and atherosclerosis are important in evaluating the cardiovascular (CV) effects and safety of antidiabetes drugs in the development of treatments targeting CV complications. Our aim was to evaluate if crossing the heterozygous glucokinase knockout mouse (GK^+/−^) and hyperlipidemic mouse deficient in apolipoprotein E (ApoE^−/−^) will generate a disease model exhibiting a diabetic and macrovascular phenotype.* Methods*. The effects of defective glucokinase on the glucose metabolism and on the progression and regression of atherosclerosis on high-fat diets were studied in both genders of GK^+/−^ApoE^−/−^ and ApoE^−/−^ mice. Coronary vascular function of the female GK^+/−^ApoE^−/−^ and ApoE^−/−^ mice was also investigated.* Results*. GK^+/−^ApoE^−/−^ mice show a stable hyperglycemia which was increased on Western diet. In oral glucose tolerance test, GK^+/−^ApoE^−/−^ mice showed significant glucose intolerance and impaired glucose-stimulated insulin secretion. Plasma lipids were comparable with ApoE^−/−^ mice; nevertheless the GK^+/−^ApoE^−/−^ mice showed slightly increased atherosclerosis development.* Conclusions*. The GK^+/−^ApoE^−/−^ mice showed a stable and reproducible hyperglycemia, accelerated atherosclerotic lesion progression, and no lesion regression after lipid lowering. This novel model provides a promising tool for drug discovery, enabling the evaluation of compound effects against both diabetic and cardiovascular endpoints simultaneously in one animal model.

## 1. Introduction

Diabetes is rising globally, with the prevalence of type 2 diabetes (T2D) especially starting to reach epidemic proportions [1]. The metabolic abnormalities that characterize T2D have long been shown to induce molecular mechanisms that contribute to accelerated atherosclerosis progression [2]. Moreover, individuals with T2D have an increased risk of developing cardiovascular diseases and higher cardiovascular mortality rates compared with individuals without T2D [3]. Despite the advances in antidiabetic treatments, effective glycemic control is not always achieved in patients and cardiovascular benefits of treatments are not always demonstrated. To address the need for antidiabetic treatments that are effective against both underlying pathology in T2D and associated cardiovascular complications, studies testing novel drug candidates require a predictive and translational animal model which closely represents the disease pathology.

In the pancreas, glucokinase (GK) determines the rate of glucose-stimulated insulin secretion (GSIS), whereas in the liver the rates of glucose utilization and glycogen synthesis are regulated by the GK activity. Thus, GK plays a critical role in the regulation of blood glucose by acting as the glucose sensor in both pancreas and liver [4, 5]. In humans, heterozygous point mutations in the glucokinase (GK) gene result in reduced enzymatic activity and decreased insulin secretion, causing maturity onset diabetes of the young (MODY) with early-onset and persistent hyperglycemia [6]. Reduced GK activity has been suggested to contribute to impaired insulin secretion, as well as to the abnormalities in hepatic glucose balance in humans with T2D [7–10].

Animal models of GK depletion have been developed in the past, but with limited success. *β*-cell specific GK knockout is lethal; therefore, a nonleptin dependent global heterozygous depletion of glucokinase gene was generated at AstraZeneca [11]: a model that has proven responsive to standard-of-care antidiabetic agents like metformin, sitagliptin, exendin, glipizide, and GK activators [12]. This heterozygous glucokinase knockout (GK^+/−^) mouse model has a global disruption of one allele for the GK gene, causing decreased GK activity in both pancreas and liver and leading to reduced insulin secretion and elevated blood glucose levels [11]. The GK^+/−^ mice were recently shown to back-translate the effects of various standard-of-care antidiabetic agents with different mechanism of actions at clinically translatable free-drug exposure levels [13]. Additionally, these authors also showed that, due to the stability of its diabetic phenotype, the GK^+/−^ mouse can be used for long-term safety studies of novel glucose-lowering agents [13]. These characteristics make the GK^+/−^ mouse an attractive model to be used in drug development programs for new antidiabetic agents.

In order to develop a diabetic atherosclerotic mouse model with characteristics that closely resemble the human T2D driven cardiovascular disease, we cross-bred the above-mentioned mouse model (GK^+/−^) into an apolipoprotein E (ApoE^−/−^) deficient background, to derive a heterozygous glucokinase and apolipoprotein E-knockout mouse (GK^+/−^ApoE^−/−^). In this study, we describe the generation of the GK^+/−^ApoE^−/−^ mice and characterize their phenotypes, diabetic as well as lipid. In addition, the progression and regression of the atherosclerotic disease process were compared with nondiabetic ApoE^−/−^ mice. The coronary vascular function of the female GK^+/−^ApoE^−/−^ mice was evaluated. The main goal was to accomplish a model that would be suitable for the evaluation of combined T2D and cardiovascular endpoints in drug development.

## 2. Materials and Methods

### 2.1. Animals

Animal care and experiments conform to the Directive 2010/63/EU of the European Parliament on the guidelines for the protection of animals used for scientific purposes and were approved by The Regional Animal Ethics Committee for Experimental Animals, University of Gothenburg. Studies are reported in accordance with the ARRIVE guidelines for reporting experiments involving animals [14].

Mice deficient in apolipoprotein E (ApoE^−/−^) on the C57BL/6N background (AstraZeneca, Sweden) were crossed with C57BL/6J mice with a global deletion in one glucokinase allele (GK^+/−^). Detailed description of the generation of the GK^+/−^ was given in a previously published article [11]. GK^+/−^ApoE^−/−^ colony was maintained by backcrossing to ApoE^−/−^ mice and thus the GK^+/−^ApoE^−/−^ mice gradually acquired a complete C57BL/6N genetic background. ApoE deficient (ApoE^−/−^) littermates with functional GK were used as controls in all experiments.

Male mice were housed individually and female mice in groups of five in rooms with regulated temperature, humidity, and a 12 hours' light-dark cycle (lights off 19:00). They had free access to normal Chow diet (R3; Lactamin AB, Kimstad, Sweden) and water, unless stated otherwise in substudies. Body weights were monitored weekly during the studies. Mice were sacrificed during anesthesia with 5% isoflurane, following blood withdrawal from left ventricle.

### 2.2. Characterization of Diabetic Phenotype

**Figure 1:**
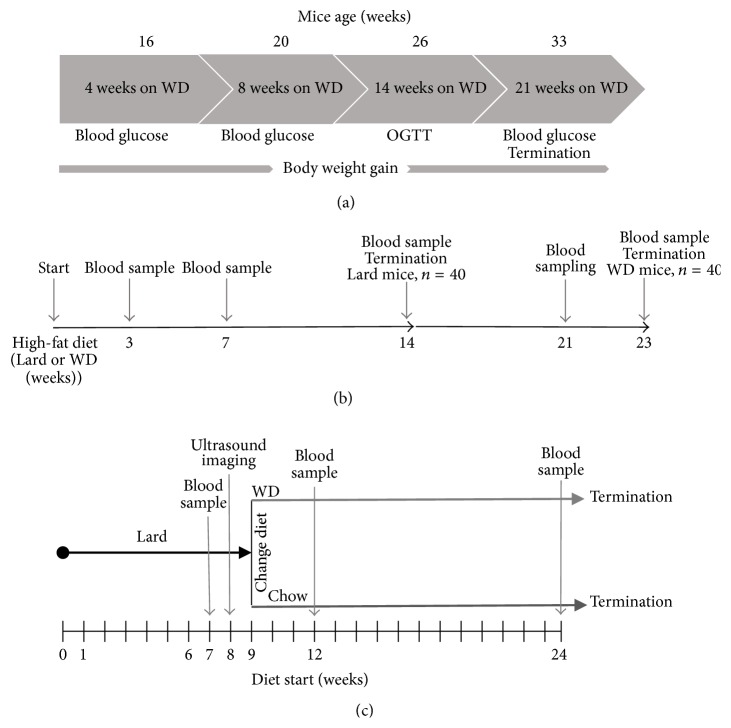
Study design for the characterization of the diabetic phenotype of the GK^+/−^ApoE^−/−^ and the ApoE^−/−^ mice on Western diet (WD) (a), study design for the atheroprogression study (b), and atheroregression study (c).

The effect of high-fat Western diet (WD) (R638; Lactamin AB, Kimstad, Sweden) on the diabetic phenotype of the GK^+/−^ApoE^−/−^ mice was investigated in 10 male and 10 female GK^+/−^ApoE^−/−^ mice compared with the sex matched wild-type ApoE^−/−^ mice (*n* = 20). Basal levels of blood glucose and insulin were measured in the mice which were fed normal Chow diet before starting the WD (Figure 1(a)). Blood glucose was measured after 4-hour fasting at 4, 8, and 14 and 21 weeks after starting WD, using a hand-held Accu-Chek glucose monitor glucometer (Accu-Chek, Roche Diagnostics, Mannheim, Germany) in the awake mice. At the same time points, a blood sample of ~20 *μ*L was collected for insulin measurement.

Oral glucose tolerance and insulin sensitivity were examined by performing a tolerance test in the GK^+/−^ApoE^−/−^ mice and the ApoE^−/−^ mice for comparison after 14 weeks on WD. Oral glucose tolerance tests (OGTT) were performed at 13:00 hour after 4-hour fasting by an oral administration of glucose (2 g/kg) and 15–20 *μ*L blood was drawn from the tail vein at 0 (right before the dosing), 15, 30, 60, and 120 min for the measurement of glucose (2 *μ*L, Accu-Chek, Roche Diagnostics, Mannheim, Germany) and insulin (2 × 5 *μ*L, Ultra-Sensitive Mouse Insulin ELISA Kit, Crystal Chemical, Downers Grove, IL) levels. Area under the curve (AUC) values were calculated for glucose and insulin as total area under the curve and corrected for differences in baseline values.

Plasma insulin levels were measured with a radioimmunoassay (SRI-13K, Millipore Corporation, USA) on a 1470 Automatic Gamma Counter (PerkinElmer, USA). Whole blood insulin was measured with an ELISA kit (Ultra-Sensitive Mouse Insulin ELISA Kit; Crystal Chem, Downers Grove, IL).

### 2.3. Atherosclerosis Progression and Regression

To investigate atherosclerosis progression on different high-fat diets, GK^+/−^ApoE^−/−^ mice and ApoE^−/−^ males and females (*n* = 10/gender/genotype) were switched from normal Chow diet to Lard diet (821424; 21% fat from pork lard, 0.15% cholesterol; Special Diets Services, Essex, UK) for 14 weeks, or Western diet (WD) (R638; 21% fat/cocoa butter, 0.15% cholesterol; Lantmännen, Kimstad, Sweden) for 23 weeks (Figure 1(b)). This aimed to accelerate the hyperlipidemia progress and pathological cardiovascular phenotype. Cholesterol was measured at 3 and 7 weeks after diet commenced and at termination (14 weeks in the Lard diet group and 23 weeks in the WD group).

In addition, to investigate atherosclerosis regression, 18-week-old GK^+/−^ApoE^−/−^ and ApoE^−/−^ males and females (*n* = 10/gender/genotype) were first transferred from Chow to Lard diet (821424; 21% fat from pork lard, 0.15% cholesterol; Special Diets Services, Essex, UK) for 9 weeks to establish atherosclerotic lesions and then split into two groups (1) Chow diet and (2) WD for 14 weeks (Figure 1(c)). Animals were allocated to the different diet intervention groups by randomization based on plaque size in the brachiocephalic artery (BCA) measured by noninvasive ultrasound imaging.

### 2.4. Analyses of Plasma Lipid Levels

Blood samples during the study were drawn from the saphenous vein and by cardiac puncture at termination, placed into MiniCollect K3E EDTA tubes (Greiner Bio-One GmbH, Kremsmünster, Austria) on ice, and centrifuged (2800 rpm, 4°C, 10 min). Plasma was stored at −80°C until analysis. Plasma lipid concentrations were measured using enzymatic colorimetric methods (total cholesterol: kit number A11A01634, Horiba ABX, France; triglycerides: kit number 12146029, Roche Diagnostics GmbH, Germany; nonesterified fatty acids: NEFA-HR (2) assay, 434-91795 and 436-91995, Wako Chemicals GmbH, Germany).

### 2.5. Histological Analyses

Heart, BCA, and thoracic aorta were dissected out and fixed in 4% phosphate-buffered formaldehyde (number 02176, HistoLab Products, Gothenburg, Sweden). Thoracic aortas were cleaned from the adventitia and opened longitudinally and macroscopic lesions were quantified* en face* and photographed and images were imported into BioPix IQ 2.1.8 image analysis system (BioPix AB, Gothenburg, Sweden) which was used for quantification. Samples of aortic sinus, ascending aorta, and BCA were embedded in paraffin and serial 4 *μ*m sections were cut (Histocenter, Gothenburg, Sweden). Sections were either stained with conventional stains (hematoxylin-eosin, Miller's elastin, or Picrosirius red; Histocenter, Gothenburg, Sweden) or immunostained for smooth muscle cells (*α*-actin, 1 : 100, clone 1A4, Dako, M0851) or macrophages (MAC-2, 1 : 10000, clone M3/38, CL8942AP, Cedarlane) using DAB (BC-BDB2004, Biocare) as the chromogen. Immunostaining was performed as single or dual stain using an automated stainer (Intellipath, Biocare). Slides were then scanned in an automated slide scanner (Mirax Scan, Zeiss) and images were analyzed with BioPix IQ 2.1.8 image analysis system (BioPix AB, Gothenburg, Sweden). Plaque area and area of positive staining were measured. Positive staining was related to plaque area. All histological procedures and analyses were performed in a blinded fashion.

### 2.6. Coronary Flow Velocity Reserve (CFVR) Measured Using Ultrasound Imaging

Transthoracic echocardiography was performed using noninvasive high-frequency ultrasound imaging in the atherosclerosis regression study on 18-week-old GK^+/−^ApoE^−/−^ and ApoE^−/−^ female mice to characterize coronary vascular function. All mice were anesthetized with Isoba® vet isoflurane (Schering-Plough Ltd., England) in a closed chamber with 3% isoflurane in oxygen for 2 to 5 minutes until immobile and 1.0–1.5% isoflurane in oxygen during the examination. Each mouse was placed supine on a heated procedure board with isoflurane initially at 1.5% supplied by a nose cone connected to the anesthesia vaporizer. Chest hair was removed with chemical cream (Veet, Reckitt Benckiser, UK). Imaging was done with a high-resolution ultrasound scanner (Vevo 770, Visualsonics Inc., Toronto, Canada) using a 40 MHz mechanical transducer with a focal depth of 6 mm. Isoflurane was reduced to 1% to lower coronary flow to a baseline level and velocity profile in the left coronary artery was measured in a modified long-axis view, recorded with a pulsed-wave Doppler for 3 minutes to ensure stable signal was achieved, after which signals were collected and stored. Isoflurane level was then increased to 2.5% to increase the coronary flow, and velocity profile was monitored for up to 4 minutes during which time signals were stored for offline analysis of maximum hyperemic response. Isoflurane was reduced back to 1%, and mice were allowed to stabilize. Detailed protocol using isoflurane to induce hyperemia has been described elsewhere [15]. Plaque size and CFVR analysis were measured offline (Vevo770 software, Visualsonics Inc., Toronto, Canada) in a blinded fashion.

CFVR was calculated as the ratio of peak diastolic flow velocities at baseline obtained using 1% isoflurane and during hyperemia obtained using 2.5% isoflurane (CFVR = hyperemic coronary flow velocity/basal coronary flow velocity).

### 2.7. Statistical Analyses

To detect a 20% difference in blood glucose at *p* < 0.05 with 80% power, we would require group size of *n* = 8. However, since detecting differences in atherosclerosis requires bigger group sizes due to expected larger variation, group sizes were *n* = 10. Numerical values for each measurement are shown as mean ± SEM. Statistical significance was evaluated using 1-sided (blood glucose, plaque size,* en face* lesion area) or 2-sided (body weight, insulin, cholesterol, and triglyceride (TG)) Student's *t*-test. Significant statistical difference was considered at *p* < 0.05. For the OGTT, ANOVA of repeated measures and Tukey's correction for multiple comparisons were used. All statistical analyses were performed using GraphPad Prism version 6.01 (GraphPad Prism version 6.01 for Windows, GraphPad Software, La Jolla, California, USA).

## 3. Results

**Figure 2:**
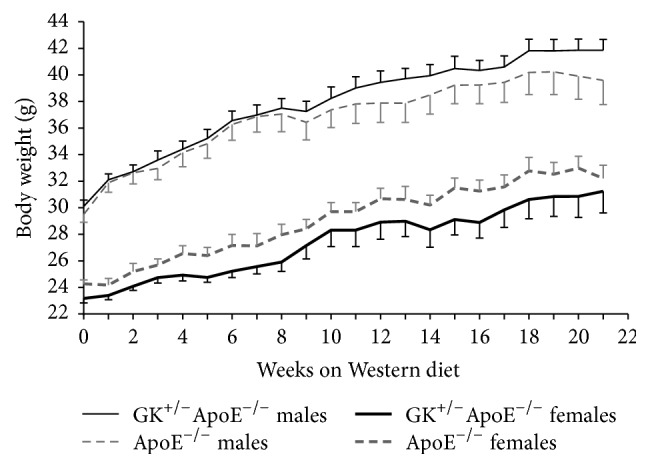
Body weight development of the GK^+/−^ApoE^−/−^ mice compared with the ApoE^−/−^ mice on WD. GK^+/−^ApoE^−/−^ males have a higher body weight compared with the ApoE^−/−^ males after 21 weeks on WD. ApoE^−/−^ females have a higher body weight from baseline Chow diet and maintained the weight difference on WD compared with GK^+/−^ApoE^−/−^ female mice. Values are presented as means and standard error of mean (SEM).

Body weight increased in both genotypes with age reflecting the growth of the animals. There were gender differences in the genotypes, where GK^+/−^ApoE^−/−^ males have a higher body weight compared with the ApoE^−/−^ males after 21 weeks on WD. Conversely, ApoE^−/−^ females have a higher body weight from baseline on Chow diet and maintained the weight difference on WD compared with GK^+/−^ApoE^−/−^ female mice (Figure 2).

### 3.1. Progression of Diabetes

#### 3.1.1. Characterization of Diabetic Phenotype

**Figure 3:**
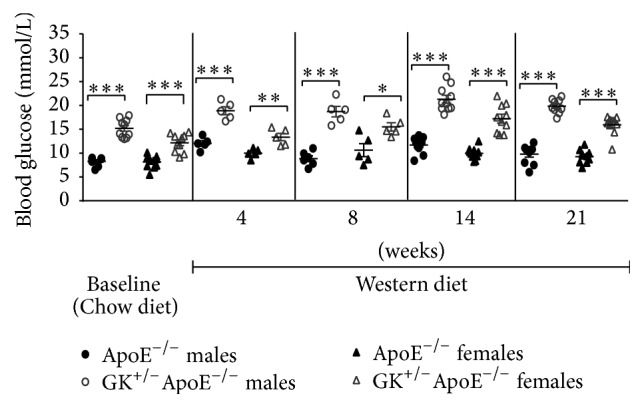
Fasting blood glucose is higher in GK^+/−^ApoE^−/−^ mice on normal Chow diet and WD at 4, 8, 14, and 21 weeks compared with ApoE^−/−^ mice. Values are presented as individual data points and means. Statistical analysis was performed using *t*-test and a *p* value of less than 0.05 was considered significant. ^*∗*^ *p* < 0.05, ^*∗∗*^ *p* < 0.005, and ^*∗∗∗*^ *p* < 0.0001.

On Chow diet, the GK^+/−^ApoE^−/−^ mice displayed significantly higher fasting blood glucose levels (15.2 ± 0.6 mmol/L in males and 12.2 ± 0.6 mmol/L in females), compared to the normoglycemic ApoE^−/−^ controls (8.3 ± 0.3 mmol/L and 8.1 ± 0.4 mmol/L in males and females, resp.) (Figure 3). When challenged with WD, the glucose levels increased steadily to a 30% higher level in the GK^+/−^ApoE^−/−^ mice (19.8 ± 0.4 mmol/L, *p* < 0.0001, in males and 15.9 ± 0.6 mmol/L, *p* = 0.0001, in females). In ApoE^−/−^ controls a significant 18% increase was seen in male mice (9.8 ± 0.6 mmol/L, *p* < 0.05), whereas in females the change was smaller and not statistically significant (15%, 9.3 ± 0.5 mmol/L, *p* = 0.1266).

**Figure 4:**
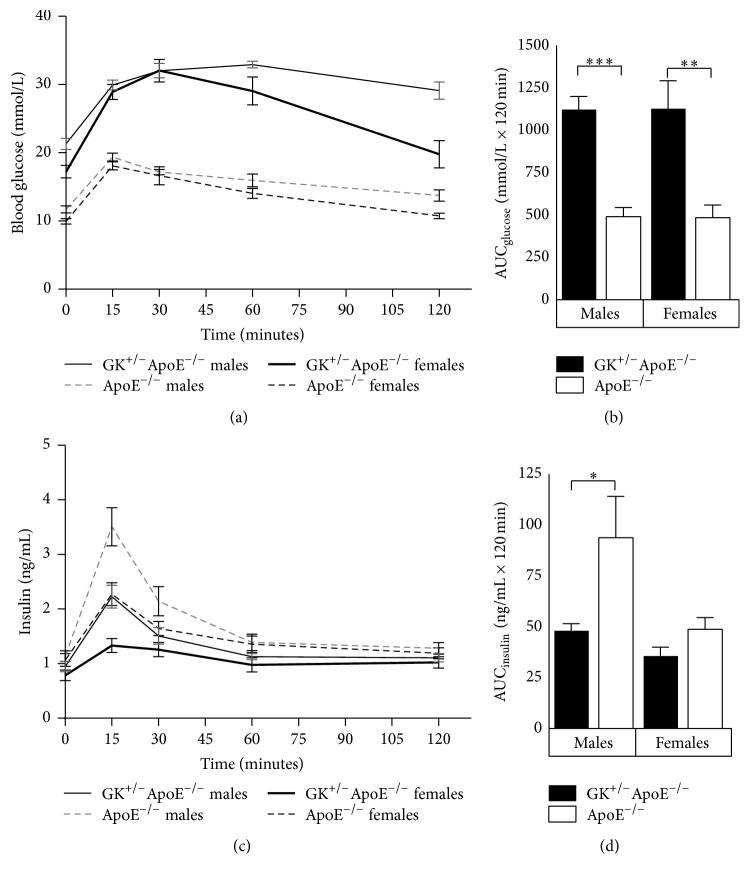
Oral Glucose tolerance test ((a) and (b)) and insulin ((c) and (d)) in the GK^+/−^ApoE^−/−^ mice compared with the ApoE^−/−^ mice after 14 weeks on Western diet. Both male and female GK^+/−^ApoE^−/−^ mice display significantly impaired glucose-stimulated insulin secretion. Values are presented as means and standard error of mean (SEM). ^*∗*^ *p* < 0.05, ^*∗∗*^ *p* < 0.005, and ^*∗∗∗*^ *p* < 0.0001.

In an oral glucose tolerance test performed after 14 weeks on WD (Figure 4), GK^+/−^ApoE^−/−^ mice showed significant glucose intolerance with 2.3-fold increased AUC_glucose_ (Figure 4(b)) and impaired GSIS with AUC_insulin_ reduced by 49% and 27% (in males and females, resp.) compared to ApoE^−/−^ mice (Figure 4(d)). Defective GSIS was reflected also in basal insulin values, which in general tended to be lower in the GK^+/−^ApoE^−/−^ than ApoE^−/−^ mice (Figure 4(c)).

#### 3.1.2. Atheroprogression and Atheroregression Studies

In the atheroprogression and atheroregression studies, hyperglycemia was very stable and more profound in GK^+/−^ApoE^−/−^ mice compared to ApoE mice (data not presented).

### 3.2. Lipids

#### 3.2.1. Atheroprogression Study

Reduction in the GK function did not affect total plasma cholesterol or triglyceride levels (Table 1). In general, cholesterol levels were higher on Lard diet compared with WD in both genotypes already after 3 weeks. GK^+/−^ApoE^−/−^ male mice have a trend for lower cholesterol from study start and show a declining tendency with time. The triglyceride levels were stable in GK^+/−^ApoE^−/−^ mice despite different diets, and there was a trend for lower values in GK^+/−^ApoE^−/−^ mice compared to ApoE^−/−^ mice (Table 1).

#### 3.2.2. Atheroregression Study

Priming with Lard diet for 9 weeks in the atherosclerosis regression study induced a 3-fold increase in total cholesterol and these levels remained the same after changing to WD with a similar cholesterol content (0.15%). On the contrary, after switching from Lard to Chow diet, the total cholesterol levels decreased significantly (43–55%) in all groups.

### 3.3. Atherosclerosis

#### 3.3.1. Atheroprogression

**Figure 5:**
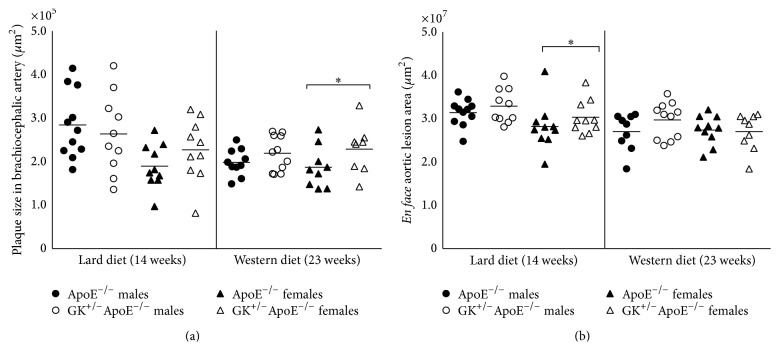
Histology evaluation of plaque size in the brachiocephalic artery (a) and* en face* lesion area in the thoracic aorta (b) measured in the atheroprogression study. There is a trend for GK^+/−^ApoE^−/−^ mice to have greater plaque compared with the ApoE^−/−^, with female GK^+/−^ApoE^−/−^ having a more consistent pattern. Statistical analysis was performed using *t*-test and a *p* value of less than 0.05 was considered significant. ^*∗*^ *p* < 0.05.

On WD, in the BCA the female GK^+/−^ApoE^−/−^ had the largest plaques (230112 ± 20124) versus female ApoE^−/−^ (188057 ± 15816) *p* = 0.0587 (Figure 5(a)). In the ascending aorta, there was a trend for GK^+/−^ApoE^−/−^ mice to have larger lesions with less variability compared with ApoE^−/−^ (Figure 5(b)). However, statistically significant difference was seen only with female mice on Lard diet (GK^+/−^ApoE^−/−^ versus ApoE^−/−^, 2602000 ± 403800 versus 1355000 ± 297600, *p* = 0.0115) (males on Lard: GK^+/−^ApoE^−/−^ versus ApoE^−/−^  4284000 ± 615660 versus 3201000 ± 465241, *p* = 0.0858).

**Figure 6:**
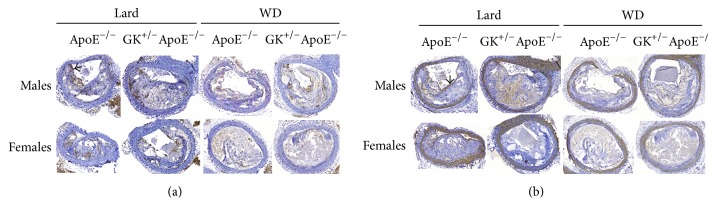
The atherosclerotic lesions of the brachiocephalic artery in both genotypes have become advanced and complicated after Lard and WD for 14 and 23 weeks, respectively. Large areas of the lesions consist of necrotic core and cholesterol clefts. (a) Only a few scattered macrophages (brown indicated by arrow on the figure) are detected by the macrophage antibody. (b) Smooth muscle cells (brown) are detected in the vessel wall by the alpha-actin antibody. A thin cap is sometimes seen in the lesions, indicated by arrow on the figure. Images in Figures 6(a) and 6(b) are consecutive sections from the same animal.

On lard diet, there was also a trend in BCA for female GK^+/−^ApoE^−/−^ to have the largest plaque (228298 ± 22675) versus female ApoE^−/−^ (190804 ± 16158), *p* = 0.0974. There were no statistically significant differences in plaque size in the brachiocephalic artery (WD 220188 ± 12279 versus 199578 ± 9759, Lard 264854 ± 28916 versus 286013 ± 23494) on either high-fat diets for the male group (GK^+/−^ApoE^−/−^ versus ApoE^−/−^). Lesion was equally advanced in the brachiocephalic artery in the two strains illustrated in Figure 6.

#### 3.3.2. Atheroregression

**Figure 7:**
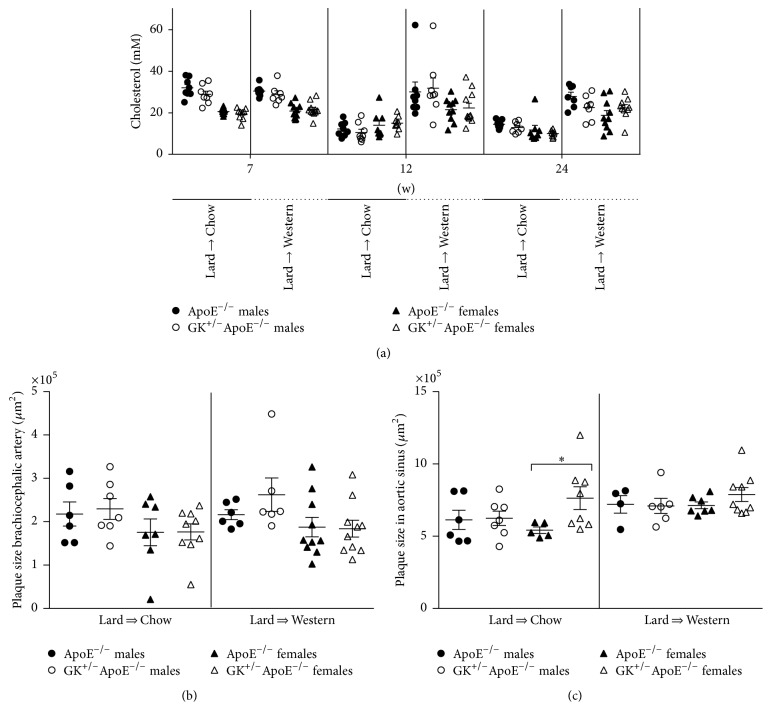
Nonfasting total plasma cholesterol measured after 7 weeks on Lard diet, 4 weeks after diet changed from Lard to WD or Lard to Chow, and 14 weeks after diet changed from Lard to WD or Lard to Chow (a). Plaque size in the brachiocephalic artery (b) and aortic sinus plaque size (c) measured after 14 weeks on Lard to WD or Lard to Chow diet in the atheroregression study. (a) Priming mice with Lard diet induced a 3-fold increase in total cholesterol and the levels remained the same after changing to WD, resulting in similar cholesterol levels at 7 weeks. Conversely, after switching from Lard to Chow diet, the total cholesterol levels decreased significantly in all groups. (b) Plaque sizes in the brachiocephalic artery in both diet groups were comparable, which indicates that the atherosclerosis did not regress on the Lard to Chow diet change. (c). Female GK^+/−^ApoE^−/−^ mice show equal amount of plaque in the aortic sinus in the Lard to Chow group compared to the Lard to WD despite lower plasma cholesterol levels in both GK^+/−^ApoE^−/−^ and ApoE^−/−^ mice after dietary change (Lard to Chow diet). Statistical analysis was performed using *t*-test and a *p* value of less than 0.05 was considered significant. ^*∗*^ *p* < 0.05.

Despite lower plasma cholesterol levels in both males and females GK^+/−^ApoE^−/−^ and ApoE^−/−^ mice after dietary change (Lard to Chow diet) compared with the Lard to WD (Figure 7(a)), plaque size in the brachiocephalic artery (Figure 7(b)) and in the aortic sinus (Figure 7(c)) remained comparable. In other vascular sites, there was also impaired lesion regression (*en face* lesion area in thoracic aorta, ascending aorta, and left coronary artery) (data not presented).

### 3.4. Coronary Flow Velocity Reserve (CFVR)

Basal flow velocity and hyperemic flow velocity (cm/s) were not significantly different in either genotype. Furthermore, CFVR was not significantly different between ApoE^−/−^ and GK^+/−^ApoE^−/−^ at 18 weeks of age (Table 2).

## 4. Discussion

The present study characterized heterozygous glucokinase knockout apolipoprotein E deficient mice (GK^+/−^ApoE^−/−^), a novel mouse model of diabetes and atherosclerosis. We demonstrate that this mouse model has very stable hyperglycemia, indications of increased atherosclerosis development on high-fat diet, and impaired lesion regression after lipid lowering. The atherosclerosis is stable on WD and the GK^+/−^ApoE^−/−^ knockout shows comparable coronary vascular function to the apolipoprotein E (ApoE) deficient mice at 18 weeks of age.

For a rodent model to be considered T2D model, fasting blood glucose levels of around 150–300 mg/dL^−1^ (8.3–16.7 mmol/L) are recommended [16]. Our model has glucose levels which exceed the minimum threshold and should therefore be considered as a diabetic model. There are other commonly used animal models of T2D (leptin-mutated ob/ob, leptin-receptor deficient db/db mice, and male missense mutated leptin-receptor Zucker Diabetic Fatty (ZDF) rats); however, they have a wide but unstable hyperglycemic range [12, 17]. In the fasted state, insulin levels were similar between the GK^+/−^ApoE^−/−^ knockout mice and the ApoE^−/−^ mice despite higher glucose levels in the GK^+/−^ApoE^−/−^. Higher glucose levels in the GK^+/−^ApoE^−/−^ would be expected to result in even higher insulin levels. This however was not the case, indicating that the hyperglycemia in the GK^+/−^ApoE^−/−^ knockout mice is predominantly due to inadequate compensatory insulin secretory response, possibly as a result of impaired *β*-cell function, an important determinant of type 2 diabetes [18, 19]. Insulin level measurement is an acceptable measure to indicate *β*-cell function in rodent models [20]; our findings are consistent with insulin response pattern in humans with advanced type 2 diabetes, where there is an insufficient insulin secretion to meet demand [19].

The GK^+/−^ mouse model has been suggested to be a representative model of type 2 diabetes, which encompasses both the hepatic and the *β*-cell GK deficiencies found to occur in the disease [11]. On a high-fat diet, the GK^+/−^ mouse has reduced islet GK activity and defects in glucose-stimulated insulin secretion [11, 21]; our current diabetic phenotype data on GK^+/−^ApoE^−/−^ knockout mice are comparable to those previously published on the GK^+/−^ by above-mentioned authors. This indicates that the hepatic and the *β*-cell GK deficiencies in the parent GK^+/−^ mice when cross-breed with the ApoE^−/−^ were transferred to the offspring GK^+/−^ApoE^−/−^ knockout mice.

The ApoE^−/−^ is a widely used mouse model of atherosclerosis [22]. Plasma lipid levels were comparable between the GK^+/−^ApoE^−/−^ and the ApoE^−/−^ mice, but nevertheless the GK^+/−^ApoE^−/−^ mice showed slightly higher plaque burden in line with atherosclerotic progression in human T2D [23], as well as impaired lesion regression at different vascular sites. Even though the plaque burden is slightly higher in the GK^+/−^ApoE^−/−^ compared to the ApoE^−/−^, both genotypes develop advanced and complicated lesions. There are only few detectable macrophages and thin protecting caps, if any. On the other hand, both genotypes have necrotic cores, cholesterol clefts, buried caps, and layered phenotype, hallmarks of complicated mice lesions [24]. Several clinical studies have shown in populations with atherosclerosis that dietary intervention may reduce the progression of the atherosclerotic plaque development [25, 26] and regression mediated by weight loss may even occur [27]. Furthermore, there are also data suggesting that coronary atherosclerosis may be regressed by diet and comprehensive lifestyle changes [28, 29]. For studies using animal models to investigate treatments targeting cardiovascular disease in diabetes, it is important that diabetic and atherosclerotic disease is stable.

This mouse model showed comparable coronary vascular function to the apolipoprotein E (ApoE) deficient mice at 18 weeks of age. Although there were no significant differences between the GK^+/−^ApoE^−/−^ and the ApoE^−/−^ mice for coronary vascular function, values presented here for basal flow velocity and CFVR measured in both genotypes are higher and lower, respectively, when compared to published data for C57Bl/6J-lep^ob^ and age matched lean litter mates (+/?) of comparable age [30]. Furthermore, in a study by Hartley et al. [15], they showed that old ApoE^−/−^ (2 years of age) mice have a higher baseline flow velocity and lower CFVR when compared to the ApoE^+/+^ wild-type mice of similar age, which is in agreement with our findings [15]. Baseline coronary flow is based on cardiac metabolic demand (hemoglobin content, oxygen saturation, and baseline hemodynamics) [31, 32], and it is reasonable to assume that the high blood glucose and cholesterol in the GK^+/−^ApoE^−/−^ and the ApoE^−/−^ mice will negatively influence their hemoglobin content, thereby increasing metabolic demand reflected by higher basal flow velocity. Correlation between CFVR and coronary atherosclerosis has been shown in mice [33]; furthermore, CFVR is reduced in atherosclerotic mice [32, 33]. In agreement, we observed low CFVR values in our mice model at 18 weeks of age and comparable plaque burden in the GK^+/−^ApoE^−/−^ and the ApoE^−/−^ mice, supporting the lack of difference in the CFVR between the strains and emphasizing macrovascular burden to underlie the coronary vascular dysfunction. In man, CFVR is used to evaluate the severity of different cardiac pathologies and values ranging from 2.5 to 5.0 have been reported, with >3.0 used as the lower limit of normal [31]. Validation studies on the GK^+/−^ApoE^−/−^ are currently ongoing within our lab to characterize changes in coronary vascular function over time (mice from 6 weeks of age) using ultrasonography and to investigate effect of lipid lowering and standard-of-care treatments for diabetes.

The strength of the GK^+/−^ApoE^−/−^ mice lies in that it has stable hyperglycemia, and diabetes is not progressive. This makes it possible to use either young or old mice to carry out short or long studies without the disease phenotype changing with age. The ZDF rats and ob/ob or db/db mice have limited age window for treatments due to rapid disease progression and large variability, which requires large group sizes. These other rodent models of T2D (ZDF rats and ob/ob or db/db mice) are monogenic models of obesity which induces hyperglycemia, which weakens their translational value as obesity is seldom caused by a monogenic mutation [17, 20]. Diabetes induced by Streptozotocin (STZ) provides a nonphysiological model, with variation dependent on STZ dose and sometimes severe adverse effect on rodents [34]. In the relatively long period of monitoring the GK^+/−^ApoE^−/−^ (up to 21 weeks), fasting blood glucose and cholesterol remained high and stable. This makes the GK^+/−^ApoE^−/−^ model suitable for investigations related to the combination of diabetes and atherosclerosis.

For decades, new diabetes drugs were approved primarily based on their glucose-lowering efficacy. However, after observations of increased cardiovascular risk with some medications, the regulatory agencies now demand studies showing that novel antidiabetic agents are at least neutral in their cardiovascular effects [35]. Although these new regulatory requirements as such apply only for clinical trials, they affect also preclinical research, as more accurate prediction of the cardiovascular effects is warranted. Secondly, instead of aiming just for cardiovascular neutrality, the future medical treatments will favor compounds that have beneficial cardiovascular effects in addition to their antidiabetic action (multifunctional compounds with hypolipidemic and antidiabetic action). Therefore, we need pharmacokinetic: pharmacodynamics (PK/PD) models, where both metabolic and cardiovascular endpoints can be studied in one and the same animal.

There are several positives in this novel animal model. The animal model has a stable hyperglycemic phenotype that is very reproducible from batch to batch, and there is little variation so that relatively small group sizes can be used. Diabetes does not cause increases in lipid levels, which is unlike most of the other used diabetes models. The etiology is rather relevant, since individuals with T2D exhibit high rates of hepatic glucose output and have reduced GK activity in liver and pancreas. These mice do not have any special health problems, so they are easy to breed and use in short- or long-term studies. The lipid profile is a negative against this model, since the ApoE^−/−^ mice are a remnant model and thus do not resemble much the human lipoprotein profile and lack also all the other functions of ApoE. Reviewing the lipid levels shows that there is no exceptional difference in the GK^+/−^ApoE^−/−^ compared with the ApoE^−/−^. Moreover, the diabetic GK^+/−^ApoE^−/−^ mice have a bit lower cholesterol and TG levels, which can be due to reduced GK activity in the liver and thus reduced lipogenesis. This is an important feature, since normally when diabetes is induced in mice, for example, with STZ or by crossing db/db mice with dyslipidemic strains, the lipid levels increase significantly and as a result supraphysiological lipid levels that easily overrun all other factors.

## 5. Conclusions

We conclude that, compared to normoglycemic ApoE^−/−^ mice, the GK^+/−^ApoE^−/−^ mice showed a stable and reproducible hyperglycemia, which induced accelerated atherosclerotic lesion progression as well as impaired lesion regression after lipid lowering. Importantly, these effects were seen without increases in plasma lipid levels. This novel model provides a promising tool for drug discovery, enabling the evaluation of compound effects against both diabetic and cardiovascular endpoints simultaneously in one animal model.

## Figures and Tables

**Table 1:** Lipid levels in GK^+/−^ApoE^−/−^ and ApoE^−/−^ mice on Lard and Western diet measured in the atheroprogression study.

Diet	Gender	Time	Genotype	Cholesterol (mmol/L)	TG (mmol/L)	NEFA
Lard	Males	3 weeks	ApoE^−/−^	38.86 ± 1.11	—	—
			GK^+/−^ApoE^−/−^	35.84 ± 1.91	—	—
		7 weeks	ApoE^−/−^	38.20 ± 1.46	—	—
			GK^+/−^ApoE^−/−^	31.00 ± 1.52	—	—
		14 weeks	ApoE^−/−^	38.32 ± 1.48	1.21 ± 0.11	0.49 ± 0.02
			GK^+/−^ApoE^−/−^	30.84 ± 1.81	0.86 ± 0.14	0.45 ± 0.02
	Females	3 weeks	ApoE^−/−^	25.88 ± 1.00	—	—
			GK^+/−^ApoE^−/−^	22.78 ± 1.73	—	—
		7 weeks	ApoE^−/−^	25.20 ± 0.97	—	—
			GK^+/−^ApoE^−/−^	23.40 ± 1.12	—	—
		14 weeks	ApoE^−/−^	20.58 ± 0.62	0.52 ± 0.13	0.55 ± 0.04
			GK^+/−^ApoE^−/−^	20.11 ± 0.82	0.27 ± 0.06	0.50 ± 0.03

Western	Males	3 weeks	ApoE^−/−^	20.84 ± 1.21	—	—
			GK^+/−^ApoE^−/−^	17.42 ± 2.47	—	—
		7 weeks	ApoE^−/−^	22.40 ± 0.75	—	—
			GK^+/−^ApoE^−/−^	18.80 ± 2.82	—	—
		21 weeks	ApoE^−/−^	31.65 ± 2.16	0.82 ± 0.09	0.39 ± 0.02
			GK^+/−^ApoE^−/−^	27.88 ± 2.78	0.81 ± 0.13	0.44 ± 0.02
	Females	3 weeks	ApoE^−/−^	17.54 ± 1.04	—	—
			GK^+/−^ApoE^−/−^	20.62 ± 1.19	—	—
		7 weeks	ApoE^−/−^	18.60 ± 0.51	—	—
			GK^+/−^ApoE^−/−^	18.40 ± 1.08	—	—
		21 weeks	ApoE^−/−^	23.35 ± 1.57	0.36 ± 0.05	0.45 ± 0.03
			GK^+/−^ApoE^−/−^	20.57 ± 2.45	0.38 ± 0.06	0.43 ± 0.03

Lard diet induced higher cholesterol levels than Western diet. There is a trend for ApoE^−/−^ mice to have higher cholesterol, triglycerides, and nonesterified fatty acids (NEFA) levels compared with GK^+/−^ApoE^−/−^. Data presented as mean ± SEM.

**Table 2:** Coronary flow velocity in female ApoE^−/−^ and GK^+/−^ApoE^−/−^ mice at 18 weeks old.

	ApoE^−/−^ (*n* = 17)	GK^+/−^ApoE^−/−^ (*n* = 14)
Basal flow velocity (cm/s)	35.1 ± 2.5	34.4 ± 2.7
Hyperemic flow velocity (cm/s)	59.1 ± 2.1	60.6 ± 2.3
CFVR	1.76 ± 0.18	1.86 ± 0.10

Data presented as mean ± SEM.
